# Correlation of Physical Sciences

**Published:** 1856-06

**Authors:** 


### EDITORIAL DEPARTMENT.

*Correlation of Physical Sciences.*—A passage in the writings of a popular author, in which he speaks, perhaps accidentally, of "physical philosophy" as "a science," thereby conveying the idea of unity and mutual dependence, has reminded us that we are too much in the habit of considering every science a thing by itself, to be studied and comprehended without reference to others.

The geologist is considered as distinct from the chemist, the chemist equally unrelated to the botanist, and so on through all the different departments of human learning, each by itself is considered as a perfect whole. The science of medicine, however, embracing as it does many collateral sciences, has a more liberal tendency. The diagnostician recognises the fact that not only the theory of sound is involved in the murmurs of a diseased lung, but that the laws of density and pressure, and of pneumatics, are also implicated.

*Specialism* in science as in disease, is a calamity. The superficial theory is that he who devotes all his time and study to a single subject, must necessarily understand it better than he who divides his hours among many studies. The notion would be a true one, were each department of disease or physics the invention and offspring of a separate intelligence, operating independently of other creative powers. But there is only one God, and He has so linked together all His works, that one theory governs the whole, while each hangs in dependence on the other. Each is a link in a chain—a thread in a net—and not an isolated, independent condition.

The effect of special study upon a very highly cultivated mind, has a curious illustration in the following circumstance. A distinguished geologist last winter delivered a course of lectures on his specialty, remarkable at once for their eloquence and sound argument. In noticing the fact that the remains of tropical plants had been found in profusion in Arctic regions, indicating a warm climate there at some early period, he endeavored to account for it by sundry hypotheses, all of them derived from his own science. If our recollection serves us he finally endorsed the notion that the earth's crust in that region, had at some period been so thin as to create a warm climate by radiation from its internal fires. This was the geological view of it, necessarily narrow and imperfect, as it was deprived of its relations to other sciences. A gentleman present explained to us, that it is well known among astronomers that at a certain definite period, far back in the world's history, the position of the earth with reference to the suns of other solar systems had been such as to cause a tropical climate in the arctic zone, and that, in the grand contra dance of the universe, we were now steadily returning to the same position, when the same conditions of climate would again ensue.

Our geologist, then, with all his learning and ingenuity, was but a specialist, incompetent to grasp the whole of his subject, because ignorant of facts in other sciences which bore directly upon it.

The pedagogue of the district school, who teaches geography to his juvenile class, sees all (to his ken) of geography in the fact that such a river seperates two hostile countries, or such a chain of mountains divides a continent in halves. To him, this, with its extended details is all. He neither teaches nor recognizes the fact that the same chain of mountains has a more important function as a reservoir of rains and a feeder of rivers; that it stands as a barrier to the moisture-laden trade-winds, and that, therefore, it has upon its one side sterile deserts, on the other unbounded luxuriance of vegetation; that to him who recognizes what may truly be called the vital functions of the globe, those mountains are typical of the ribbed walls which bound our respiration; that the sunny equator is the thorax of the earth, to which, through a vena-cava represented by the great oceanic currents, flows in the cold venous blood of the Arctics, to be warmed and vivified, and then returned through gulf-streams and similar aortas to the cold extremities again —that the frigid north wind has for its trachea the broad Atlantic valley, leading it on to the heated tropics, whence like very breath indeed, it pours back over the continents warmed and loaded with moisture.

Extend this field of observation, and we shall find that *Botany* is in this sense a mere province of geography. Whereover the warm, moist winds of the equator find their way, we find a vegetation profuse and luxuriant. Broad, expanded leaves, and juicy, succulent stems, rapid growth, short life, and rapid decomposition are the law of vegetative life. Or, if we go far inland on the continent, or far north beyond the reach of this moisture, the green and sappy luxuriance has disappeared. The tough and hardy oak usurps the pithy palm, the narrow leaved ash permits the feebler sunlight to strike through its branches to the earth. Juiciness has given place to tough and woody fibre, the ephemeral existence of the tropical plant is extended over centuries in the northern forest-tree. Decay, no longer the work of an hour, becomes the process of years.

Link in *Chemistry* here, the most self-reliant and self-sustaining of all the sciences, and we find that under this vital action, this physiological function of the earth, organic chemistry changes all its forms. All its metamorphoses are slower, its products more elaborate as we reach the dry, continental climate. The cell is smaller, water mingles less largely in the fruits — they are less diluent, more solid, and have a stronger taste and odor.

Thus have we imperfectly, and by broad generalities, only, endeavored to show that Astronomy, Geology, Physical Geography, Botany, and Chemistry, are but fruits of one branch, each dependent on the same support, and each the offspring of a common stock and not to be fully appreciated except in connection with each other. Add to them the science of man in its broader designation of "*Ethnology*," and we shall also find that the geography of a race governs its character, and that the same physical properties which mark the vegetative world, may be assigned to the animal kingdom.

Draw the broad distinction between the Eastern and Western Continents — the first a dry, unshaded land; the other teeming with moisture and buried beneath impenetrable forests. There the native man, Asiatic, African, or European, is a man of muscle and fibre, all his qualities resemble the tough and hardy plants begotten by the same dry atmosphere. Here, beneath the forests, we find only the vegetative Indian, incapable of prolonged labor or exertion, and sinking beneath the tasks imposed by the earlier emigrants, until the hardy negro from the dry uplands of Africa assumed his burden.

If in this hasty sketch of what might and must yet be done in the correlation of physical sciences, we have enforced our own convictions of the narrow and disastrous influences of specialism, we have attained our object. He who studies one science only may almost be said to strive for the *accumulation* of ignorance. So, too, in medicine, the specialist is incompetent to a knowledge of one branch of science, without a study of its correlates. Narrowness of view, bigotry and self-conceit, are the natural results of such a trammel on the intellect.

